# Mortality in Norway and Sweden during the COVID-19 pandemic

**DOI:** 10.1177/14034948211047137

**Published:** 2021-10-05

**Authors:** Frederik E. Juul, Henriette C. Jodal, Ishita Barua, Erle Refsum, Ørjan Olsvik, Lise M. Helsingen, Magnus Løberg, Michael Bretthauer, Mette Kalager, Louise Emilsson

**Affiliations:** 1 Clinical Effectiveness Research Group, Oslo University Hospital and University of Oslo, Norway; 2 Faculty of Health Sciences, The Arctic University of Norway, Norway; 3 Department of General Practice, University of Oslo, Norway; 4 Vårdcentralen Årjäng & Centre for Clinical Research, Sweden; 5 Department of Medical Epidemiology and Biostatistics, Karolinska Institute, Sweden

**Keywords:** Mortality, COVID-19, Norway, Sweden, epidemics, public health

## Abstract

*Background:* Norway and Sweden are similar countries in terms of socioeconomics and health care. Norway implemented extensive COVID-19 measures, such as school closures and lockdowns, whereas Sweden did not. *Aims:* To compare mortality in Norway and Sweden, two similar countries with very different mitigation measures against COVID-19. *Methods:* Using real-world data from national registries, we compared all-cause and COVID-19-related mortality rates with 95% confidence intervals (CI) per 100,000 person-weeks and mortality rate ratios (MRR) comparing the five preceding years (2015–2019) with the pandemic year (2020) in Norway and Sweden. *Results:* In Norway, all-cause mortality was stable from 2015 to 2019 (mortality rate 14.6–15.1 per 100,000 person-weeks; mean mortality rate 14.9) and was lower in 2020 than from 2015 to 2019 (mortality rate 14.4; MRR 0.97; 95% CI 0.96–0.98). In Sweden, all-cause mortality was stable from 2015 to 2018 (mortality rate 17.0–17.8; mean mortality rate 17.1) and similar to that in 2020 (mortality rate 17.6), but lower in 2019 (mortality rate 16.2). Compared with the years 2015–2019, all-cause mortality in the pandemic year was 3% higher due to the lower rate in 2019 (MRR 1.03; 95% CI 1.02–1.04). Excess mortality was confined to people aged ⩾70 years in Sweden compared with previous years. The COVID-19-associated mortality rates per 100,000 person-weeks during the first wave of the pandemic were 0.3 in Norway and 2.9 in Sweden. ***Conclusions:* All-cause mortality in 2020 decreased in Norway and increased in Sweden compared with previous years. The observed excess deaths in Sweden during the pandemic may, in part, be explained by mortality displacement due to the low all-cause mortality in the previous year.**

## Background

COVID-19 was declared an international health crisis by the World Health Organization (WHO) on 30 January 2020 and a pandemic on 11 March 2020 [1]. By 8 March 2021, 2,606,572 deaths had been officially recorded worldwide as a consequence of the disease [2] and the real death toll may be even higher [3]. Assessments of the burden of COVID-19 have been hampered by a lack of comprehensive data on the disease and of the benefits and harms of the measures against it [4]. Cause-specific death rates are prone to bias, especially for a disease with a high asymptomatic burden and large differences in the testing and reporting of causes of death between countries. All-cause mortality trends may therefore provide a more reliable alternative to assess the burden of a pandemic in different countries and regions [3,5].

Norway and Sweden are similar countries in terms of life expectancy, governmental and administrative systems, socioeconomics and public health care systems, and both countries have reliable, timely and complete registration of all deaths [6–9]. The first COVID-19-associated death, defined as a death among people with a positive COVID-19 test up to 30 days prior to their death, occurred in Norway on 12 March 2020 and in Sweden on 11 March 2020 [2]. Starting from 12 March 2020, both countries strongly emphasised general measures against COVID-19: social distancing, with a preferred distance of at least 1 m; a focus on hand-washing and disinfection; and self-quarantine for all people with symptoms suggestive of COVID-19. Contact tracing, isolation and follow-up of COVID-19 transmission was strictly enforced by public health authorities in both countries [10] (Table S1).

On 12 March 2020, the Norwegian government introduced extraordinary measures against the pandemic [11]. Emergency laws required the closure of all day-care centres, schools, universities and other academic institutions, as well as gyms, hair salons, restaurants and movie theatres. Domestic and international travel restrictions were introduced and all sport and cultural events, as well as all organised sports, were cancelled [11]. The Norwegian government urged the population to stay at home if possible and contact with health care services was encouraged only if absolutely necessary. Most appointments for patients with chronic diseases were cancelled or replaced with telephone or video consultations [12].

In Sweden, measures were considerably less strict during the first wave of the pandemic. On 27 March 2021, the Swedish government banned public gatherings and events with >50 people [13]. The Public Health Agency recommended high schools and universities to remain open, but to teach online if possible, and advised that non-essential travel should be avoided [14]. People with respiratory symptoms should stay at home and some elective surgery was postponed [15], but otherwise the health care service in Sweden operated as before. Restaurants and bars, gyms, hair salons and movie theatres stayed open, and sports and cultural events continued during the pandemic [16].

At the end of 2020, Norway reported few COVID-19-associated deaths and little severe disease. By contrast, Sweden reported more COVID-19-associated deaths and disease. The substantially different national strategies to control the COVID-19 pandemic in the two countries and the similarity of the countries with regard to confounding variables may provide a natural experiment enabling difference-in-difference analyses [17] to explore the possible benefits and harms associated with the pandemic and its measures. Previous studies were conducted early in the course of the pandemic before complete data for 2020 became available, or have not directly compared countries, states or regions with similar socioeconomics, infrastructure, ethnicity or health care systems [18–20].

In our ecological study, we used real-world data and transparent calculations to compare all-cause mortality and COVID-19-associated deaths in Norway and Sweden during the COVID-19 pandemic in 2020 with the years preceding the pandemic.

## Aims

To compare mortality in Norway and Sweden, two similar countries with very different mitigation measures against COVID-19.

## Methods

### Data sources

Norway and Sweden have similar, single-payer, public health care systems with universal coverage. In each country, all residents are assigned a unique national registration number, which provides information on sex and date of birth and allows linkage to national registers with data on socioeconomic characteristics, health and disease, hospitalisation and death. Because reporting of death and other health and socioeconomic variables is mandatory for all residents, national registries are close to 100% complete [6,7]. Since its emergence in February 2020, COVID-19 has been categorised as a communicable disease with mandatory, immediate reporting of all positive cases to the Norwegian Institute of Public Health in Norway and the Public Health Agency in Sweden.

### Study design

We retrieved weekly numbers of deaths (regardless of cause) and the population in Norway and Sweden from Statistics Norway [21], the National Board of Health and Welfare (Sweden) [22] and Statistics Sweden [23]. These registries are complete with three (Norway) and two (Sweden) weeks delay in registration of deaths. Weekly data are stratified according to the International Organization of Standardization 8601-week numbering, in which the week starts on Monday and week 1 of the year is the week with the year’s first Thursday in it. In both countries, the deaths registered in week 53 (only 2015 and 2020) were not included in the analyses.

### Statistics

We calculated weekly mortality rates per 100,000 person-weeks in each country from 1 January 2015 to 31 December 2020, separately for each year and the mean of 2015–2019. Mortality rates were based on the weekly number of deaths in Norway and Sweden and the total number of people living in Norway on 1 January of the current year [21] and in Sweden on 31 December of the previous year [23] – for example, the mortality for all weeks of 2020 were based on the number of people registered on 1 January 2020 in Norway and on 31 December 2019 in Sweden. We compared the year 2020 (the COVID-19 pandemic year) and each preceding year (2015–2019) with the mean of the five years before the pandemic (2015–2019). All analyses were stratified by pre-defined age groups (0–69, 70–79 and ⩾80 years) due to the association between severe COVID-19 and age.

We calculated the mortality rate ratios (MRR) by comparing the mean weekly mortality rates in the pandemic year 2020 with the five preceding years (2015–2019) separately for each country. We estimated the 95% confidence intervals (CI) for MRRs assuming the number of deaths to follow a Poisson distribution. We compared the weekly mortality rate in Norway and Sweden before (2015–2019) and after (2020) the start of the pandemic using a two-sample *t*-test and assuming unequal variance. The significance level of the *t*-test was set at 0.05 (two-sided). We then used the same *t*-test method to compare the change in the weekly mean mortality rate in each country after the start of the pandemic (2020 minus 2015–2019). The *t*-tests were repeated in each age group.

For both countries, we calculated the excess number of deaths in each age group in 2020 compared with the mean for 2015–2019. The excess number of deaths was calculated by first subtracting the MRR of each age group (2020 versus the mean of 2015–2019) from the reference (i.e. 1) and then multiplying the resulting number by the mean number of deaths in 2015–2019.

In separate analyses, we estimated cause the specific COVID-19 mortality rates in Norway and Sweden by retrieving information on all COVID-19-associated deaths in 2020 from 12 March in Norway and 11 March in Sweden until 22 January 2021 from the Institute of Public Health in Norway [24] and the Public Health Agency of Sweden [25], stratified by age group (0–69, 70–79 and ⩾80 years). COVID-19-associated deaths are defined as a death among people with a positive COVID-19 test up to 30 days prior to their death.

We calculated the all-cause mortality rates associated with the first wave of the pandemic and included only the corresponding weeks (weeks 12–30; 16 March–26 July 2020) and compared these weeks with the same week numbers in the preceding years. We used Stata 16.1 (StataCorp, College Station, TX, USA) for all analyses.

### Ethics

All data analysed in this report are publicly available and therefore no ethical approval was required.

## Results

### Study population

The populations in Norway and Sweden increased slightly from 2015 to 2020, from 5,165,802 in Norway and 9,747,355 in Sweden in 2015 to 5,367,580 in Norway and 10,327,589 in Sweden in 2020. The age distributions in the two populations were similar in 2020: 88% of the inhabitants of Norway and 85% of the inhabitants of Sweden were 69 years or younger; 8 and 10% of the inhabitants were aged 70–79 years in Norway and Sweden, respectively; and 4 and 5% of the inhabitants were aged ⩾80 years in Norway and Sweden, respectively.

### All-cause mortality

**Figure 1.**
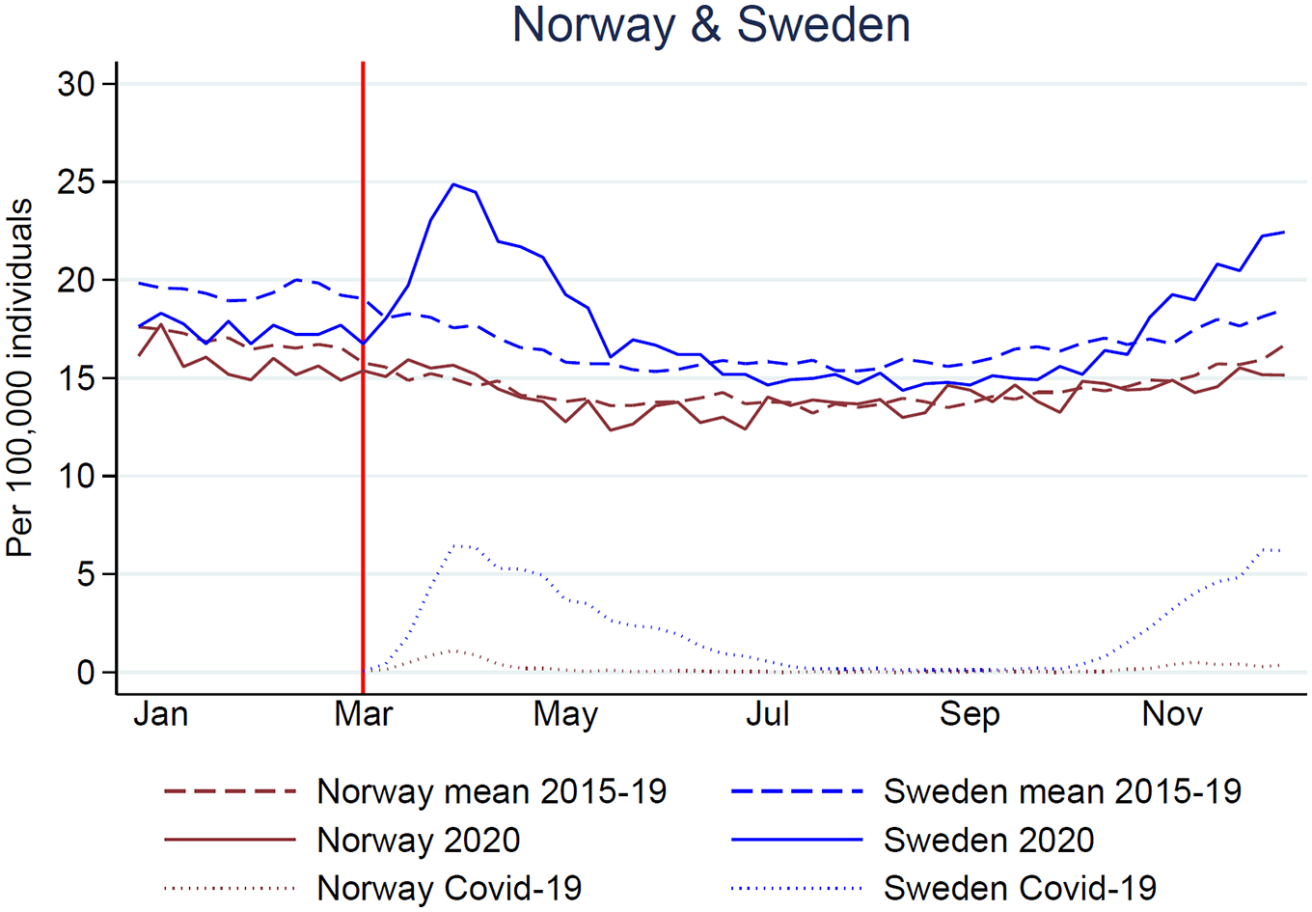
All-cause mortality rates per 100,000 person-weeks in Norway (brown) and Sweden (blue) for 2020 (solid lines), mean 2015–2019 (dashed lines) and COVID-19-associated mortality rates (dotted lines). The red vertical line shows the time point for the first COVID-19-associated deaths in Norway and Sweden (11 and 12 March 2020).

The mean number of deaths per week varied between 773 (2020) and 783 (2018) in Norway and between 1653 (2019) and 1817 (2020) in Sweden (Table I). In Norway, the all-cause mortality rates (per 100,000 person-weeks) were stable from 2015 to 2019 (mortality rate 14.6–15.1; mean mortality rate 14.9) and lower in 2020 compared with 2015–2019 (mortality rate 14.4; MRR 0.97; 95% CI 0.96–0.99) (Table I; Figures 1 and S1–S3). In Sweden, all-cause mortality rates (per 100,000 person-weeks) were stable from 2015 to 2018 (mortality rate 17.0–17.8; mean mortality rate 17.1) and similar in 2020 (mortality rate 17.6), but lower in 2019, the year immediately preceding the pandemic (mortality rate 16.2). Compared with the years 2015–2019, the all-cause mortality in the pandemic year was 3% higher (MRR 1.03; 95% CI 1.02–1.04) due to the 8% lower mortality rate in 2019 compared with 2020. Weekly mean mortality rates were significantly lower in Norway than in Sweden, both before and after the start of the pandemic (*p* < 0.01) (Table S2). In addition, the change in the weekly mean mortality rate within each country in 2020, compared with 2015–2019, was significantly different in Norway (*p* = 0.04), but not in Sweden (*p* = 0.10).

**Table I.** Weekly mean number of deaths and mortality rates per 100,000 person-weeks (2015–2020) and mortality rate ratios comparing the preceding years 2015–2020 with the mean of 2015–2019.

	No. of deaths		Mortality rate			Each year compared with mean of 2015–2019			
	Mean per week	Range	MR	CI (95%)	Range	Difference in mortality rate	CI (95%)	MRR	CI (95%)
***Norway***									
Mean 2015–2019	781	(663–1 048)	14.9	(14.7–15.0)	(12.7–19.9)	Ref.		Ref.	
2020	775	(662–952)	14.4	(14.1–14.7)	(12.3–17.7)	−0.4	(−0.6 to −0.3)	0.97	(0.96–0.98)
2019	778	(677–914)	14.6	(14.3–14.9)	(12.7–17.2)	−0.3	(−0.4 to −0.1)	0.98	(0.97–0.99)
2018	783	(671–950)	14.8	(14.4–15.2)	(12.7–17.9)	−0.1	(−0.2 to 0.1)	0.99	(0.98–1.01)
2017	781	(688–1048)	14.9	(14.4–15.3)	(13.1–19.9)	0.0	(−0.2 to 0.2)	1.00	(0.99–1.01)
2016	778	(669–982)	14.9	(14.5–15.3)	(12.8–18.8)	0.1	(−0.1 to 0.2)	1.00	(0.99–1.01)
2015	782	(663–977)	15.1	(14.7–15.6)	(12.8–18.9)	0.3	(0.1–0.4)	1.02	(1.01–1.03)
***Sweden***									
Mean 2015–2019	1709	(1442–2204)	17.1	(16.9–17.3)	(14.1–21.8)	Ref.		Ref.	
2020	1819	(1484–2569)	17.6	(16.8–18.4)	(14.4–24.9)	0.5	(0.4–0.6)	1.03	(1.02–1.04)
2019	1653	(1443–1946)	16.2	(15.8–16.5)	(14.1–19.0)	−0.9	(−1.1 to −0.8)	0.94	(0.94–0.95)
2018	1720	(1442–2204)	17.0	(16.4–17.6)	(14.2–21.8)	−0.1	(−0.2 to 0.0)	0.99	(0.99–1.00)
2017	1728	(1489–2156)	17.3	(16.8–17.8)	(14.9–21.6)	0.2	(0.1–0.3)	1.01	(1.00–1.02)
2016	1712	(1466–2033)	17.4	(17.0–17.8)	(14.9–20.6)	0.3	(0.1–0.4)	1.02	(1.01–1.02)
2015	1732	(1466–2114)	17.8	(17.3–18.3)	(15.0–21.7)	0.7	(0.5–0.8)	1.04	(1.03–1.05)

CI: confidence interval; MR: mortality rate; MRR: mortality rate ratio.

**Figure 2.**
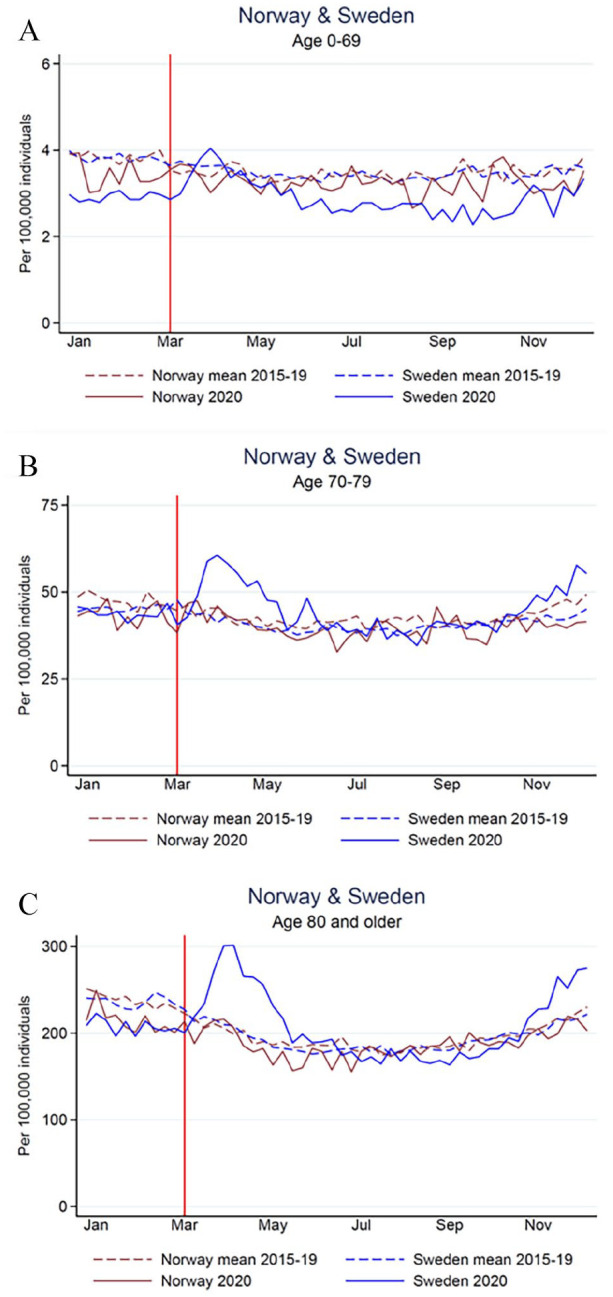
All-cause mortality rates per 100,000 person-weeks in age groups (A) 0–69 years, (B) 70–79 years and (C) ⩾80 years in Norway (brown) and Sweden (blue) for 2020 (solid lines) and mean 2015–2019 (dashed lines) and COVID-19-associated mortality rates (dotted lines). The red vertical line shows the time point for the first COVID-19-associated deaths in Norway and Sweden (11 and 12 March 2020).

For all age groups in Norway, all-cause mortality rates in 2020 were lower than the mean of the five preceding years (Table II; Figures 2(A–C) and S4(A–C)) and lower or similar to each separate year (Table S3). In Sweden, the age group 0–69 years had a lower mortality rate in 2020 than in previous years (MRR 0.82; 95% CI 0.81–0.84), whereas the older age groups had an increased all-cause mortality rate compared with previous years (70–79 years: MRR 1.07; 95% CI 1.06–1.09; ⩾80 years: MRR 1.03; 95% CI 1.02–1.04) (Table II). The all-cause mortality rate for Swedes aged ⩾80 years in 2020 was similar to the rates in 2015 and 2017 (Table S3). Before the pandemic year, mortality rates were significantly different in Norway and Sweden for the age group 70–79 years only (*p* < 0.01), whereas the country differences were significant for all age groups in 2020 (*p* ⩽ 0.01). The change in weekly mean mortality rates in 2020 compared with 2015–2019, were significantly different in all age groups (*p* < 0.01).

**Table II.** Weekly number of deaths and mortality rates per age group per 100,000 person-weeks in 2020 and the mean of 2015–2019 and mortality rate ratios comparing 2020 with the mean of 2015–2019.

	2020				Mean 2015–2019				2020 compared with mean of 2015–2019	
	No. of deaths		Mortality rates		No. of deaths		Mortality rates			
	Mean	Range	MR	CI (95%)	Mean	Range	MR	CI (95%)	MRR	CI (95%)
***Norway***										
Age 0–69 years	155	(125–185)	3.3	(3.2–3.4)	165	(123–218)	3.5	(3.5–3.6)	0.93	(0.91–0.95)
Age 70–79 years	177	(143–210)	40.6	(39.5–41.5)	164	(119–212)	43.5	(43.0–44.1)	0.94	(0.91–0.96)
Age ⩾80 years	443	(357–573)	193	(187–198)	451	(358–663)	203	(200–206)	0.95	(0.93–0.96)
***Sweden***										
Age 0–69 years	256	(200–356)	2.9	(2.8–3.0)	303	(230–386)	3.5	(3.5–3.6)	0.82	(0.81–0.84)
Age 70–79 years	442	(342–599)	44.7	(42.9–46.4)	380	(299–491)	41.7	(41.3–42.1)	1.07	(1.06–1.09)
Age ⩾80 years	1121	(876–1617)	209	(199–219)	1027	(803–1421)	202	(199–205)	1.03	(1.03–1.04)

CI: confidence interval; MR: mortality rate; MRR: mortality rate ratio.

### COVID-19 associated mortality

From 11 March 2020 to 22 January 2021, a total of 60,896 people tested positive for COVID-19 and 544 COVID-19 cause-specific deaths were recorded in Norway compared with 547,166 COVID-19 positive people and 11,005 COVID-19 cause-specific deaths in Sweden. The age distributions of COVID-19 cause-specific deaths were 14.7% (0–69 years), 20.4% (70–79 years) and 64.9% (⩾80 years) in Norway and 8.9% (0–69 years), 20.9% (70–79 years) and 70.2% (⩾80 years) in Sweden (Table S4). The COVID-19 cause-specific mortality rate during the first wave of the pandemic was 0.3 per 100,000 person-weeks (95% CI 0.1–0.4) in Norway and 2.9 per 100,000 person-weeks (95% CI 1.9–3.9) in Sweden (Table S5).

### Mortality during the first pandemic wave

During weeks 12–30, the all-cause mortality rate was 13.9 per 100,000 person-weeks (95% CI 13.4–14.5) in Norway, 0.3 fewer deaths per 100,000 person-weeks compared with the mean for 2015–2019. In Sweden, the all-cause mortality rate was 18.7 per 100,000 person-weeks (95% CI 17.1–20.3), 2.3 more deaths per 100,000 person-weeks compared with the mean for 2015–2019 (Table S5). Using the countries’ population in 2020 as a reference, this corresponds to 16 fewer deaths in Norway and 238 more deaths in Sweden per week over a 19-week period.

## Discussion

Our study shows that, in Norway, the all-cause mortality rates in 2020 were lower than or similar to the five preceding years for all age groups. In Sweden, the all-cause mortality rates in 2020 were similar to or higher than in previous years for people older than 70 years, but lower for people younger than 70 years. The COVID-19-associated mortality rate was almost ten-fold higher (2.9 versus 0.3 per 100,000 person-weeks) in Sweden than in Norway and the peaks of COVID-19 cause-specific deaths corresponded to the observed peaks in all-cause mortality (Figure 1).

The Swedish strategy against COVID-19 has received international attention and criticism, notably because reported COVID-19 mortality rates in Sweden have been higher than in comparable countries such as Norway. The similarity of Norway and Sweden with regard to COVID-19 risk factors, socioeconomics and demographics, life expectancy and comorbidity, governmental and administrative systems, health care service, education and other potential confounding variables [6–9] provided an interesting case study to explore whether there are signs that the more intense mitigation measures in Norway and the less intense measures in Sweden may have contributed to the countries’ mortality patterns.

In Sweden, mortality was lower than expected in the year preceding the pandemic. The number of COVID-19-associated deaths far exceeds the excess all-cause mortality, indicating that some of the COVID-19 cause-specific deaths occurred among vulnerable people who might have died of other causes had it not been for the pandemic. These findings may suggest mortality displacement as part of the excess mortality in 2020 in Sweden. Mortality displacement [26] entails temporarily increased mortality (called excess mortality) in a population as a result of external events, such as heat waves [27] or pandemics such as influenza [28] or COVID-19. The observed temporary excess mortality probably arises because people in vulnerable groups die weeks or months later or earlier than they would otherwise as a result of the timing and severity of the unusual external event. The excess mortality is therefore preceded and/or followed by periods of mortality that are lower than expected. The period preceding the excess mortality in Sweden during the COVID-19 pandemic was characterised by a gradual decrease in mortality from 2015 to 2019 and was particularly low in 2019. Compared with the mean of the years 2015–2018, 2019 had >3600 less deaths in total, which, according to the theory, might have increased the number of vulnerable people when the pandemic hit in 2020.

After the COVID-19 pandemic, we might see a decrease in mortality below normal levels in Sweden because the oldest and frailest have already died. Indeed, the mean remaining life expectancy in Norway and Sweden were similar before the pandemic (16.6 years in people aged 70 years and 9.4 years in people aged 80 years in 2019), which means that a large proportion of the elderly die within a few years [23]. Similarly, Norway may see an increase in mortality because the oldest and frailest have lived longer than they would have without the pandemic. As the pandemic is still ongoing, it is too early to observe this decrease in Sweden, or increase in Norway, and to reliably estimate the total effect of the pandemic.

We observed a mortality that was lower than expected for the age group 0–69 in both countries through the pandemic year compared with each of the five preceding years. Hence, for the working population that comprises >85% of the two countries, the COVID-19 pandemic has not had a negative impact on all-cause mortality. The reason for this may be due to basic measures against COVID-19, such as social distancing, personal hygiene, self-quarantine for people with symptoms and contact tracing, which were applied in both countries. However, the decrease in mortality rate in the age group 0–69 years in Sweden in the pandemic year was significantly larger than in Norway.

The fight against COVID-19 required extensive health care resources [11,29] and studies from other countries indicate that morbidity and mortality from causes other than COVID-19 have changed after the outbreak of the pandemic [30]. As the people in nursing homes are old and frail, >50% of COVID-19-related deaths during the pandemic occurred in these homes [31]. However, the number of deaths in Swedish nursing homes is higher than in Norway and our study shows that the mortality rates in older age groups increased significantly more in Sweden than in Norway. A government-appointed commission in Sweden has stated that the country failed to protect elderly people and some have pointed out that health care staff in Swedish nursing homes were more likely to show up at work sick because of a lack of sick leave compensation for workers [31,32]. In addition, there were few health staff in Swedish nursing homes and many of the employees were part-time workers.

Another difference between Norway and Sweden that may have contributed to the differences in mortality is the proportion of self-employed general practitioners in primary care: about 95 and 20%, respectively [9]. Although primary health care is central in both countries, this might have affected how the pandemic was handled. For example, in Norway, every inhabitant is appointed to a personal general practitioner (*fastlege*) and there are twice as many general practitioners per inhabitant in Norway than in Sweden [9]. These general practitioners were central in contact tracing and the isolation of infected people during the first year of the pandemic.

A limitation of this study is that we relied on the cause of death from the registries for COVID-19-associated mortality, which is dependent on the registration of COVID-19 among those who died. A Swedish study reviewing the medical records of 122 people (a total of 51% of all deaths in the study region) found that 70% of the deaths were COVID-19-associated; however, only 15% of the deaths were identified as directly caused by the virus [33]. COVID-19 may have contributed to the other 85% of deaths registered as COVID-19-associated, but heart, lung or other diseases were the main cause of death. Hence the proportion of COVID-19 deaths in our study may be an overestimation.

The observational study design does not provide an opportunity to draw conclusions about the causal relationship between the mitigation measures, either as a total or for specific measures (e.g. school-closing), in Norway and Sweden and their effects on the two countries’ differences in mortality rates. Sweden’s chief epidemiologist has pointed out that Sweden has a higher proportion of non-European immigrants than Norway and the incidence of COVID-19 has been higher in these immigrants than in the general population in both countries since the start of the pandemic [34,35]. In addition, the population in Norway is slightly younger than in Sweden [21,23], which may contribute to our finding of a difference in mortality rates after the start of the pandemic. It is possible that the more intensive measures implemented in Sweden following the country’s second pandemic wave may add to our understanding of which measures influence all-cause mortality in the future.

This is the first study to compare complete 2020 real-world data from two very similar countries, but with different overall approaches to control the pandemic. This provides an interesting setting for our difference-in-difference analysis, investigating the association of mitigation measures with mortality, without the use of modelling and by using transparent calculations. The registration of all-cause mortality is mandatory and similar in Norway and Sweden, making all-cause mortality in the two countries more reliable for assessing the burden of the COVID-19 pandemic than cause-specific deaths only, which can be biased by differences in the testing and reporting of causes of death.

This study shows that all-cause mortality in Norway was lower during the pandemic, whereas the all-cause mortality among elderly people in Sweden increased substantially. In previous years, both countries have seen a decreasing trend in all-cause mortality. It remains to be seen whether the observed excess deaths in Sweden during the pandemic may, in part, be explained by mortality displacement and whether the COVID-19 pandemic and mitigation measures are associated with other harms or benefits.

## Supplemental Material

sj-docx-1-sjp-10.1177_14034948211047137 – Supplemental material for Mortality in Norway and Sweden during the COVID-19 pandemic Click here for additional data file. Supplemental material, sj-docx-1-sjp-10.1177_14034948211047137 for Mortality in Norway and Sweden during the COVID-19 pandemic by Frederik E. Juul, Henriette C. Jodal, Ishita Barua, Erle Refsum, Ørjan Olsvik, Lise M. Helsingen, Magnus Løberg, Michael Bretthauer, Mette Kalager and Louise Emilsson in Scandinavian Journal of Public Health
