# Supplementary material for: Mortality in Norway and Sweden during the COVID-19 pandemic
Source: Scand J Public Health. 2021 Oct 5;50(1):38–45. doi: 10.1177/14034948211047137 (PMC8807990; doi:10.1177/14034948211047137)
Supplement: sj-docx-1-sjp-10.1177_14034948211047137 – Supplemental material for Mortality in Norway and Sweden during the COVID-19 pandemic [file sj-docx-1-sjp-10.1177_14034948211047137.docx]

**Supplementary figures and tables**

**Mortality in Norway and Sweden during the Covid-19 Pandemic**

FE Juul, HC Jodal, I Barua, et al.

**Content:**

Supplementary figures

- *Figure S1:* All-cause mortality rates in Norway and Sweden in 2020, the five preceding years, and covid-19 associated mortality rates.
- *Figure S2:* Number of deaths in Norway and Sweden in 2020, the mean of 2015-2019, and covid-19 associated number of deaths.
- *Figure S3:* Number of deaths in Norway and Sweden in 2020, the five preceding years, and covid-19 associated deaths.
- *Figure S4:* Number of deaths in age groups 0-69 years, 70-79 years and ≥ 80 years in Norway and Sweden in 2020 and the mean of 2015-2019.

Supplementary tables

- *Table S1: Covid-19 mitigation measures in Norway and Sweden in 2020.*
- *Table S2: Statistical comparison of weekly mean mortality rates per 100,000 person-weeks in Norway vs Sweden.*
- *Table S3:* Weekly number of deaths, mortality rates per age group, per 100,000 person-weeks for years 2015-20, and mortality rate ratios comparing years 2015-20 to the mean of 2015-19.
- *Table S4:* Excess all-cause deaths in 2020 compared to the mean of 2015-19 and Covid-19 associated deaths in 2020.
- *Table S5:* Number of deaths and mortality rates during first pandemic wave (weeks 12-30).

**Figure S1: All-cause mortality rates per 100,000 person-weeks in Norway (A) and Sweden (B) in 2020 (coloured lines), the five preceding years (grey lines), and covid-19 associated mortality rates (dotted). The red vertical line shows the time point for the first covid-19 associated death in Norway and Sweden (March 11 and 12).**


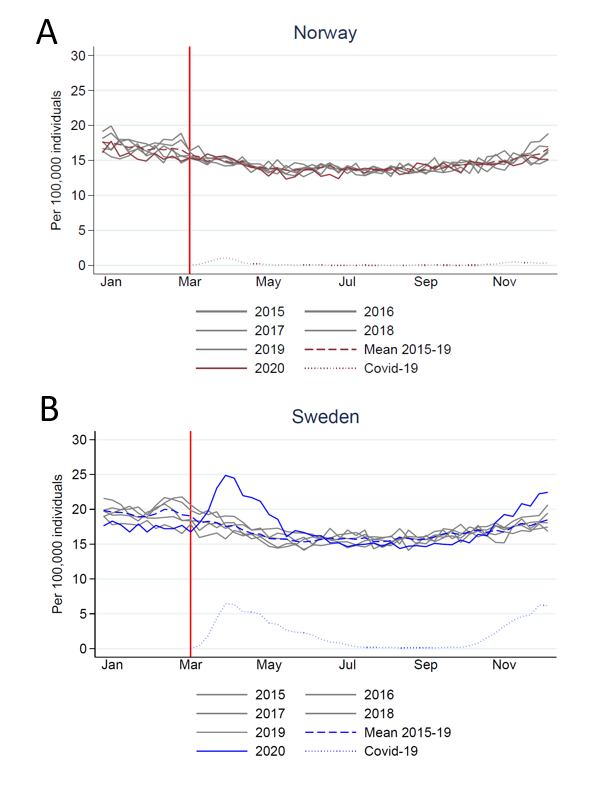


***Figure S2:* Number of deaths in Norway (brown) and Sweden (blue) in 2020 (solid lines), the mean of 2015-2019 (dashed lines), and covid-19 associated number of deaths (dotted). The red vertical line shows the time point for the first covid-19 associated death in Norway and Sweden (March 11 and 12).**


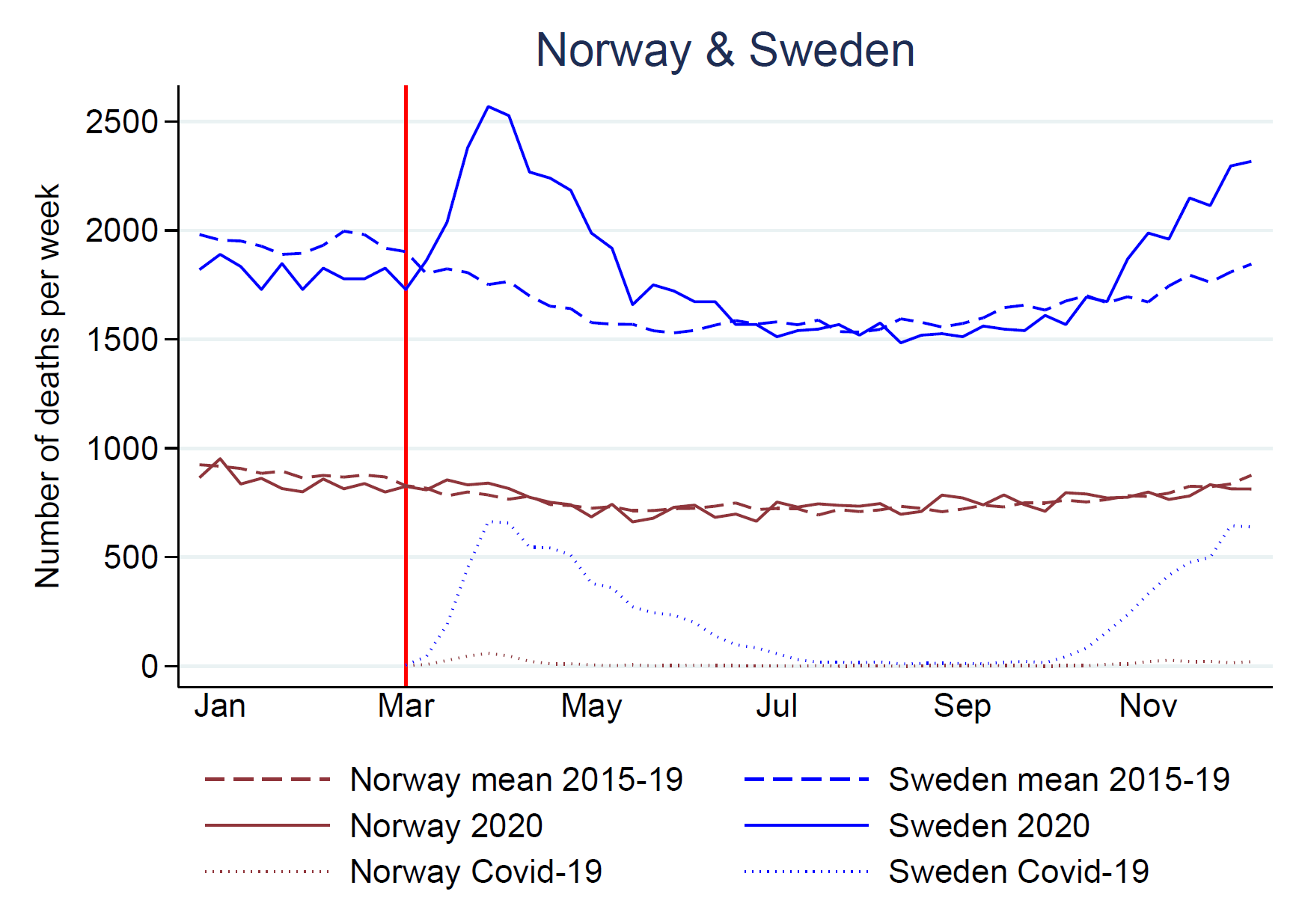


***Figure S3:* Number of deaths in Norway (A) and Sweden (B) in 2020 (coloured lines), the five preceding years (grey lines), and covid-19 associated deaths (dotted). The red vertical line shows the time point for the first covid-19 associated death in Norway and Sweden (March 11 and 12).**


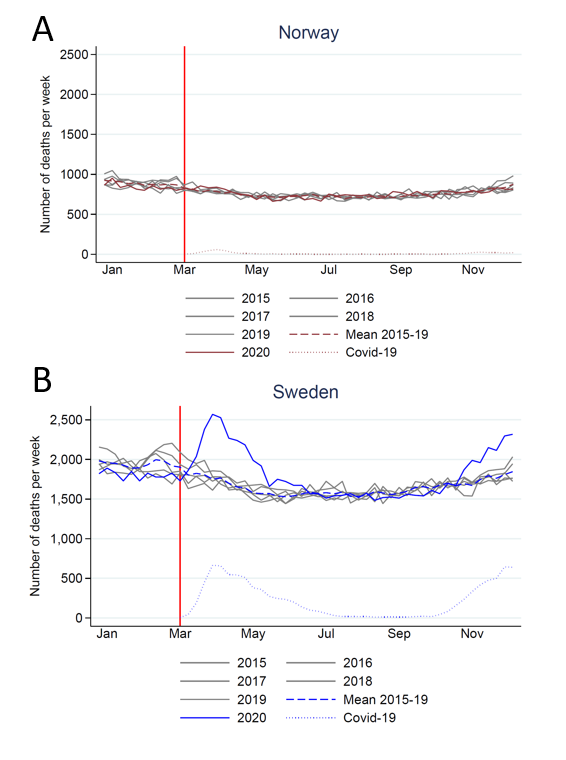


***Figure S4:* Number of deaths in age groups 0-69 years (A), 70-79 years (B) and ≥ 80 years (C) in Norway (brown) and Sweden (blue) in 2020 (solid lines) and the mean of 2015-2019 (dashed lines). The red vertical line shows the time point for the first covid-19 associated death in Norway and Sweden (March 11 and 12).**


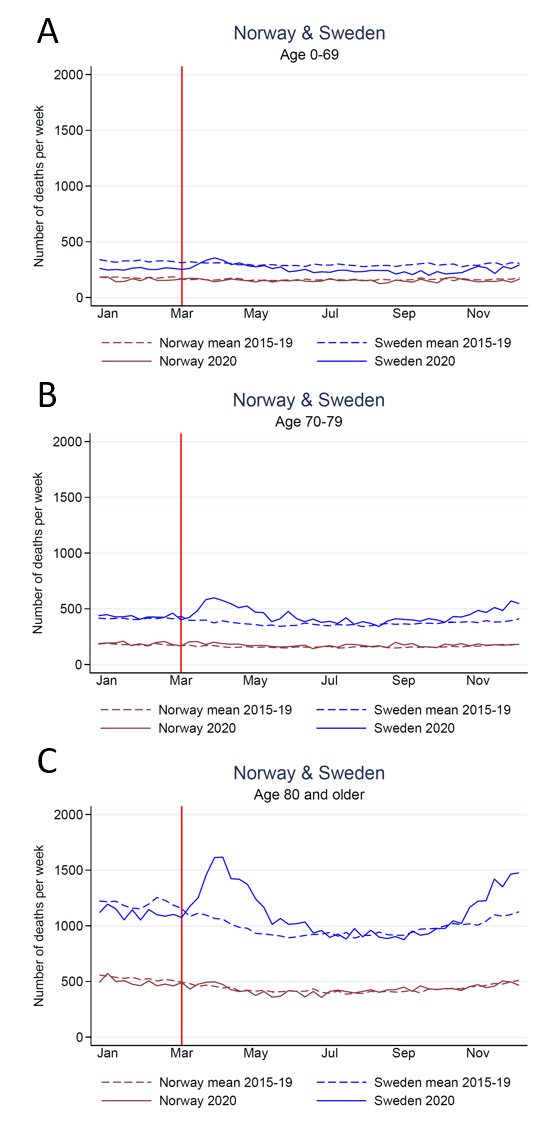


***Table S1:* Covid-19 mitigation measures in Norway and Sweden in 2020.**

*References for measure description and dates*

*Norway:* Timeline: News from Norwegian Ministries about the Coronavirus disease Covid-19*,* [www.*regjeringen.no*](http://www.regjeringen.no)*, accessed 14 May 2021*

*Sweden:* The first eight months of Sweden’s COVID‐19 strategy and the key actions and actors that were involved*, J.F. Ludvigsson, September 2020, doi.org/10.1111/apa.15582;* [*https://www.folkhalsomyndigheten.se/*](https://www.folkhalsomyndigheten.se/) *(accessed 14 May 2021)*

**Table S2: Statistical comparison of weekly mean mortality rates per 100,000 person-weeks in Norway vs Sweden.**

Using two sample t-test, assuming unequal variance.

*CI: confidence interval; MR: mortality rate*

**Table S3: Weekly number of deaths, mortality rates per age group, per 100,000 person-weeks for years 2015-20, and mortality rate ratios comparing years 2015-20 to the mean of 2015-19.**

*CI: confidence interval, MR: mortality rate; MRR: Mortality rate ratio*

**Table S4: Excess all-cause deaths in 2020 compared to the mean of 2015-19 and Covid-19 associated deaths in 2020.**

Negative numbers indicate lower number of deaths in 2020, compared to mean 2015-19. Cumulative number of Covid-19 associated deaths in Norway and Sweden from 11 March 2020 (first death in Sweden) and to 22 January 2021.

**Table S5: Number of deaths and mortality rates during first pandemic wave (weeks 12-30).**

Number of deaths and MRs are weekly mean per 100,000 person-weeks, in 2020 and mean of the same weeks in 2015-19. MR difference and MRR compare 2020 to the mean of 2015-19.

*CI: confidence interval, MR: mortality rate, MRR: Mortality rate ratio*
